# Neonatal cortical astrocytes possess intrinsic potential in neuronal conversion in defined media

**DOI:** 10.1038/s41401-020-00586-0

**Published:** 2021-02-05

**Authors:** Peng Zeng, Qiu-hong Hua, Jun-yuan Gong, Chang-jie Shi, Xiao-ping Pi, Xin Xie, Ru Zhang

**Affiliations:** 1 Shanghai Key Laboratory of Signaling and Disease Research, Laboratory of Receptor-based Bio-medicine, Collaborative Innovation Center for Brain Science, School of Life Sciences and Technology, Tongji University, Shanghai, 200092 China; 2 CAS Key Laboratory of Receptor Research, the National Center for Drug Screening, Shanghai Institute of Materia Medica, Chinese Academy of Sciences, Shanghai, 201203 China

**Keywords:** cortical astrocytes, neonatal, neuronal conversion, insulin, heterogeneity

## Abstract

Astrocytes are multifunctional brain cells responsible for maintaining the health and function of the central nervous system. Accumulating evidence suggests that astrocytes might be complementary source across different brain regions to supply new neurons during adult neurogenesis. In this study, we found that neonatal mouse cortical astrocytes can be directly converted into neurons when exposed to neurogenic differentiation culture conditions, with insulin being the most critical component. Detailed comparison studies between mouse cortical astrocytes and neuronal progenitor cells (NPCs) demonstrated the converted neuronal cells originate indeed from the astrocytes rather than NPCs. The neurons derived from mouse cortical astrocytes display typical neuronal morphologies, express neuronal markers and possess typical neuronal electrophysiological properties. More importantly, these neurons can survive and mature in the mouse brain in vivo. Finally, by comparing astrocytes from different brain regions, we found that only cortical astrocytes but not astrocytes from other brain regions such as hippocampus and cerebellum can be converted into neurons under the current condition. Altogether, our findings suggest that neonatal astrocytes from certain brain regions possess intrinsic potential to differentiate/transdifferentiate into neurons which may have clinical relevance in the future.

## Introduction

Neurogenesis is usually robust during early brain development, but drops rapidly during adulthood. New neurons can derive from neural stem cells and/or neural progenitor cells (NPCs), however, these cells become very limited in aging brains. Besides, NPCs are restricted mainly in two brain zones, the subventricular zone (SVZ) and the subgranular zone (SGZ) of the dentate gyrus of the hippocampus, which are usually considered as canonical neurogenic regions in adult mammalians [1]. Other brain areas, such as the striatum [2], cortex [3], and hypothalamus [4], have been shown to possess neurogenic abilities, and the source of new neurons outside of the canonical neurogenic regions remains elusive.

Astrocytes are the most abundant type of cells distributed over almost every region of the brain. Astrocytes are known to have a wide variety of physiological functions, including the regulation of neurotransmitter and ion concentrations, the production of trophic factors and the maintaining of blood-brain barrier [5, 6]. Besides, astrocytes were implied as important regulators to control neural stem cell proliferation and their commitment to a neuronal fate [7]. Recently, many studies focus on neurogenesis by conversion of post-mitotic astrocytes into neuronal cells which provides alternative strategies for modeling neurological diseases and regenerative medicine [8–11]. These studies suggest that epigenetic “reprogramming,” either by forced expression of transcription factor or treatment of chemical compounds can drive the conversion of astrocytes to neuronal cells [10]. Other reports suggest that astrocytes might possess intrinsic neuronal conversion potential. It has even been speculated that glial cells might be a kind of NPC-like cells due to the observations that astrocytes can express certain NPC markers such as Nestin and Sox2, whereas certain glial markers such as GFAP has also seen in NPCs [12–15]. When astrocytes became reactive in response to injury or environment stressors, they can re-enter the cell cycle and subsequently dedifferentiate and reprogram into neurons [16, 17]. In the adult mammalian hippocampus, astrocytes have been seen to give rise to new neurons even under normal conditions [12]. Thus, the neurogenic property of astrocytes is considered to be a promising way to overcome the limitation of endogenous neurogenesis from NPCs which are restricted in certain niches and the disadvantages of external cell transplantation, to supply new neurons during aging and degenerative diseases throughout the brain. Indeed, astrocytes from different brain regions including cortex, cerebellum and spinal cord have been recently chosen as the initial cells for neuronal reprogramming [18, 19]. However, the influence of astrocytes heterogeneity on their neuronal conversion ability is not clearly elucidated.

Herein we found that neonatal mouse cortical astrocytes can be directly converted into neurons when exposed to a widely used neuronal differentiation culture condition. Further studies revealed that the neuronal conversion ability of neonatal mouse cortical astrocytes is distinct from that of neonatal NPCs. The neuronal conversion of cortical astrocytes can be fulfilled by a simple supplement of insulin in the medium. Furthermore, the astrocytes from other brain regions such as hippocampus and cerebellum do not possess this direct conversion ability. These results provide more insights into the neurogenic ability of astrocytes and enrich our knowledge of the region-specific features of astrocytes.

## Materials and methods

### Animals

For lineage tracing experiments, GFAP-Cre mice (Jackson Laboratory, stock number J012886) were mated with R26RtdTomato mice (Jackson Laboratory, stock number J007905) to generate mice with specific expression of tdTomato in astrocyte, Aldh1L1::GFP mice generated astrocytes carrying Aldh1L1::GFP reporter. Specific pathogen-free grade mice were housed in the animal facility of Tongji University, Shanghai, China. All animal maintenance and experimental procedures were performed in accordance with the Tongji University Guide for the Use of Laboratory Animals.

### Cell cultures

Primary astrocytes were isolated from day 1, day 7 or day 14 postnatal mouse brain as previously described [20]. Mouse pup was sacrificed by decapitation using the scissors after being sprayed with 70% ethanol. The brain is taken out and placed into dissecting dish filled with HBSS on ice, the next dissection procedures were performed under a stereomicroscope. The olfactory bulbs and the cerebellum were removed using the fine dissecting forceps and then peel away the plate-like structure of the cortex from the brain, finally dissect the meninges from the cortex hemispheres carefully by pulling with the fine forceps. Cortex was cut into small pieces and transferred into the Falcon tube and digested with 0.25% trypsin in water bath at 37 °C for 30 min, shaking every 10 min. and then treated with 0.4% DNaseI (Worthington, LS002007) at 37 °C for 5 min. Vigorous pipetting was performed using a 10 mL plastic pipette until tissue pieces are dissociated into single cells, cells are filtered using 40 μm filter gauze, centrifuged at 300 ×*g* for 5 min and the supernatant was decanted. The dissociated single cell was resuspended using astrocyte medium (DMEM/F12, Gibco, 11330032) supplemented with 10% FBS (Hyclone, SH30084), 2% B27 (Gibco, 17504044), 10 ng/mL bFGF (PeproTech, 100-18B), 10 ng/mL EGF (PeproTech, 100-47) and 1% penicillin/streptomycin (Millipore, TMS-AB2-C) and the suspension was plated on poly-*D*-lysine (Sigma-Aldrich, P0899) coated dishes and incubated at 37 °C in the CO_2_ incubator for one week. The medium was changed every 2 days, dishes were shaken after reaching confluence and then astrocytes were digested and cryopreserved in liquid nitrogen.

For E14.5 NPCs or day 1 postnatal NPCs isolation, mouse pup was sacrificed by decapitation using the scissors after being sprayed with 70% ethanol. The brain is taken out and placed into dissecting dish filled with HBSS on ice, the next dissection procedures were performed under a stereomicroscope. The olfactory bulbs and the cerebellum were removed using the fine dissecting forceps and then the plate-like structure of the cortex was peeled away from the brain, finally the meninges were dissected from the cortex hemispheres carefully by pulling with the fine forceps. Cortex was grinded to single cells in 40 μm filter gauze using Syringe handle, the cells were filtered using 40 μm filter gauze, centrifuged at 300 ×*g* for 5 min and the supernatant was decanted. Single cells were resuspended using NPC medium (DMEM/F12 supplemented with 2% B27, 20 ng/mL bFGF, 20 ng/mL EGF, 1% Glutamax (Gibco, 35050061)) and incubated at 37 °C in the CO_2_ incubator for about 3 days, neurospheres were digested and cryopreserved in liquid nitrogen.

For primary neurons culture, single cells were resuspended using neuron culture medium (Neurobasal supplemented with 2% B27, 1% Glutamax, 5 pg/mL plasmocin and 1% penicillin/streptomycin), plated on dishes, and incubated at 37 °C in the CO_2_ incubator, 2 days later, 1 μM Arac (Sigma, C6645) was added into the neuron culture medium, the medium was changed every 2 days, and neurons were cultured for 7 days and were used for the following experiment.

### Induction of neuronal cells from astrocytes or NPCs

Astrocytes were resuscitated and plated on poly-*D*-lysine coated dish and cultured with astrocyte medium for 3 days, cells were digested and plated on poly-*D*-lysine coated coverslips with astrocyte medium overnight. NPCs were resuscitated and cultured with NPC medium for 3 days, neurospheres were digested and plated on poly-*D*-lysine and laminin coated coverslips with NPC medium overnight. For subsequent induction, the medium was changed to neuronal induction medium (DMEM/F12 supplemented with 2% B27, 1% N2 (Gibco, 17502048), 20 ng/mL BDNF (PeproTech, 450-02), 20 ng/mL GDNF (PeproTech, 450-10). The medium was changed every 2 days. For particular experiment, astrocytes were transferred into B27 and N2 containing medium (DMEM/F12 supplemented with 2% B27 or 1% N2) or DMEM/F12 medium supplemented with corresponding insulin (5 μg/mL), transferrin (0.1 mg/mL), sodium selenite (5.2 ng/mL), progesterone (6.3 ng/mL) and putrescine·2HCl (16.11 μg/mL).

### Co-culture with primary neurons

Mouse primary postnatal cortical neurons were isolated and cultured for about one week before re-plating the induced cells. After astrocytes were treated in induction medium for 4 days, the induced neuronal cells were dissociated by using 0.00625% trypsin and centrifuged for 5 min at 200 ×*g* at room temperature. Cells were resuspended with neuron culture medium and were re-plated into pre-existing primary neurons, the re-plated cells were co-cultured for about 10 days or longer, and the cells were subjected to electrophysiology test.

### Immunofluorescence staining

Cells cultured on coverslips were fixed with 4% paraformaldehyde for 15 min at room temperature. After washed with PBS for 3 times and incubated in blocking and permeabilization buffer (2% BSA and 0.3% TritonX-100 in PBS), cells were then incubated with primary antibody at 4 °C overnight, fluorescent probe-conjugated species-specific secondary antibodies and DAPI were used to stain the cells at room temperature for 1 h. The brain tissues were post-fixed with 4% PFA for 24 h, allowed to settle in a 30% sucrose solution for 48 h and were frozen in liquid nitrogen for 10 min. Brain tissue was sectioned at 30 μm thickness, sections were washed with PBS for 3 times and blocked with PBS buffer containing 5% BSA and 0.5% Triton X-100 for 1 h at room temperature. And then the sections were incubated with primary antibody at 4 °C overnight and stained with fluorescent probe-conjugated species-specific secondary antibodies and DAPI (1:1000). Primary antibodies were as follows: DCX (1:500, abcam, ab18723); Tuj1 (1:1000, covance, PRB435P); MAP2 (1:1000, abcam, ab32454); NeuN (1:200, Millipore, ABN78); GAD67 (1:200, Millipore, MAB5406); Vglut1 (1:200, Millipore, MAB55021); TH (1:200, Millipore, AB152); CHAT (1:200, Millipore, AB144P); Syn1 (1:500, Millipore, AB1543); GFAP (1:200, DAKO, Z0334); S100β (1:200, Sigma, S2532); Nestin (1:200, covance, MAB353); Sox2 (1:200, Santacruz, SC17320); NG2 (1:200, Millipore, AB5320); MBP (1:500, abcam, ab7349). The secondary antibodies (1:1000) were as follows: Goat anti-Rabbit Alexa Fluor 488 (Invitrogen, A11034); Goat anti-Rabbit Alexa Fluor 546 (Invitrogen, A11010); Goat anti-Mouse Alexa Fluor 488 (Invitrogen, A11001); Goat anti-Mouse Alexa Fluor 546 (Invitrogen, A11003); Goat anti-Rabbit Alexa Fluor 647 (Invitrogen, A21245); Goat anti-Mouse Alexa Fluor 647 (Invitrogen, A21236); Goat anti-Rat Alexa Fluor 488 (Invitrogen, A11006); Goat anti-Rat Alexa Fluor 546 (Invitrogen, A11081); Donkey anti-Goat Alexa Fluor 488 (Invitrogen, A11055).

Slides and sections images were captured using Olympus IX71 and Fluoview FV10i. The number of single- or double-stained cells was counted using Image Pro-Plus software. For every individual experiment, we selected one or more slides for staining and captured 5 to10 visual fields(1.45 mm^2^) randomly for each slide. data were statistically analyzed using graphpad prism 5.

### Quantitative real-time PCR

For quantitative real-time PCR, the total RNA was isolated from cultured cells with TRIzol (Roche) following manufacturer’s instructions. cDNA was generated from 1 μg of total RNA with the random hexamers and M-MLV reverse transcriptase (Promega). Quantitative real-time PCR was performed with primers and 2**×** SYBR Green qPCR Master Mix (selleck, B21702) in an MX3000P Stratagene PCR machine. The quantity of sample was calculated using the standard curve method, the relative expression values were normalized against the internal controls, and then we take neuron group as control, the data was graphed using graphpad prism 5. The primers are shown in Supplementary Table S1.

### Electrophysiology

As previously described [21], patch clamp recording was performed at room temperature on astrocytes derived tdTomato neurons after co-culture with primary neurons for 14 days. The Giga-Ohm seal was achieved under the voltage-clamp mode and the APs were recorded under the current-clamp configuration using an Axopatch-200B amplifier (Molecular Devices). Signals were analyzed with pClamp10.4 software.

### Fluorescence-activated cell sorting

Day 1, day 7 and day 14 postnatal tdTomato or GFP positive astrocytes were digested and washed with PBS for three times and resuspended with PBS containing 2% BSA. Cells were then sorted with Beckman flow cytometry (Moflo Astrios), sorting threshold was setup based on tdTomato and GFP negative wt astrocytes.

### Cell transplantation in vivo

tdTomato positive astrocytes were treated in induction medium for 4 days, the induced neuronal cells were dissociated by using 0.00625% trypsin and resuspended at a density of 3 × 10^3^ cells/μL in cold neuron culture medium. Cell suspensions were injected into the lateral ventricles of day 1 postnatal mouse pups, with 1 µL injected into each hemisphere.

### Statistic analysis

All quantified data were statistically analyzed and presented as average ±SEM. Two-tailed Student *t*-tests were used to calculate statistical significance with *P*-values, unless otherwise stated. *P*-values less than 0.05 (*P* < 0.05) were considered indicative of significance.

## Results

### Conversion of neonatal astrocytes into neuronal cells by a defined medium

Previous studies have shown that mouse brain astrocytes can be converted into neuronal cells by either chemical cocktail VCR (Valproic acid, Chir99021, and Repsox) or transcription factors such as NeuroD1 or Ngn2 [8, 22, 23]. To further investigate the phenomenon, we used a lineage-tracing experiment to track the origin of the neuronal cells. The R26RtdTomato mice harboring a loxP-flanked STOP cassette preventing transcription of a DsRed fluorescent protein (tdTomato) were crossed with transgenic mice carrying the Cre recombinase gene driven by an astrocyte glial fibrillary acidic protein (GFAP) promoter. Therefore, the progeny of these mice (GFAP-Cre:R26RtdTomato) would have the tdTomato expressed in the astrocytes and progeny cells derived from astrocytes.

**Fig. 1:**
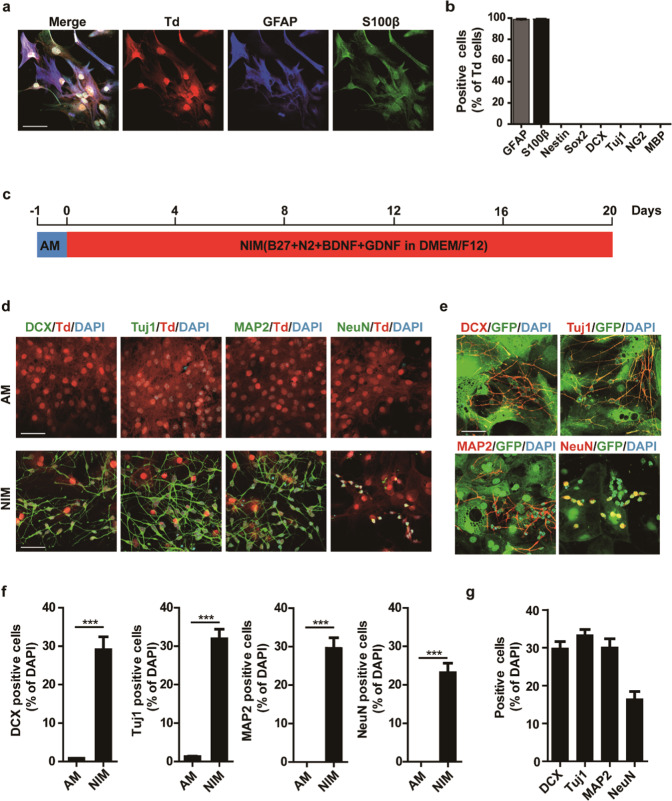
Induction of neuronal cells from mouse astrocytes by induction medium. **a** Cultured cells were tdTomato (Td) positive and expressed astrocyte markers GFAP and S100β. **b** Quantification of **1b** and **S1a** (mean ± SEM, *n* = 3 independent experiments). **c** Schematic diagram showing the process of neuronal induction. AM, astrocyte growth medium; NIM, neuronal induction medium. **d** Immunostaining of tdTomato positive cells in AM or NIM on day 8 with DCX, Tuj1, MAP2 antibodies and on day 12 with a NeuN antibody. **e** Immunostaining of GFP positive cells cultured in NIM with DCX, Tuj1, MAP2 antibodies on day 8 and on day 12 with a NeuN antibody. **f**, **g** Statistic analysis of **d** and **e**. Quantification of Neuronal cells derived from tdTomato positive cells (**f**) or GFP positive cells on day8 (**g**) (mean ± SEM, *n* = 3 independent experiments). ***P < 0.001. Scale bars, 50 μm

The neonatal astrocytes were isolated and immunofluorescence analysis suggested that more than 95% of the cells expressing tdTomato were also positive for astrocytic markers GFAP and S100β (Fig. 1a, b). In addition, the isolated cells were negative for NPC marker Nestin and Sox2, neuronal marker DCX and Tuj1, oligodendrocyte precursor cell (OPC) marker NG2, and oligodendrocyte marker MBP in immunofluorescence staining (Fig. 1b, Supplementary Fig. S1a). qPCR analysis confirmed that these cells expressed high level of *GFAP*, *Aldoc*, *GS* and *CD44*, but not *Nestin* and *Sox2* or *Tuj1 *and *NeuN*, which were highly expressed in E14.5 NPCs and primary neurons, respectively (Supplementary Fig. S1d-k). These results suggest that the tdTomato-positive cells represent the astrocytes properly.

To study chemical-mediated astrocyte to neuron reprogramming, tdTomato-positive astrocytes isolated from neonatal cortex were cultured in the astrocyte culture medium (AM) for 1 day, and then switch to neuronal induction medium (NIM) with or without a chemical cocktail VCR [8] (Fig. 1c). To our surprise, we found that the conversion of astrocytes to neuronal cells could be achieved simply by switching the culture medium to NIM without extra chemical addition (Supplementary Fig. S2 and Supplementary Video 1). After 2-day culture in NIM, astrocytes changed their morphology clearly and bipolar neuron-like cells could be observed after 4-day culture. Furthermore, about 30% of the tdTomato-positive cells were immunopositive for neuronal markers including DCX, Tuj1 and MAP2 on day 8, and 23% of the tdTomato-positive cells were immunopositive for NeuN on day 12 (Fig. 1d, f and Supplementary Fig. S3). In contrast, when astrocytes were maintained in primary astrocyte culture condition, no change in cell morphology was observed and neuronal markers were hardly detected on day 8 or even later (Fig. 1d, upper panel and 1f). The proportion of DCX positive cells was 0.8%, for Tuj1 positive cells it was 1.3%, and for MAP2 and NeuN positive cells it was 0% in AM.

ALDH1L1 is another specific marker for astrocytes, neonatal astrocytes from ALDH1L1:GFP transgenic mice expressing GFP under the control of the ALDH1L1 promoter were also tested. GFP-positive astrocytes were isolated from the cortex of ALDH1L1:GFP mice and purified with flow cytometry. Immunostaining revealed that approx. 98% of the GFP-expressing cells were positive for astrocytic marker GFAP and S100β and were negative for the NPC marker Nestin and Sox2, neuronal marker DCX and Tuj1, OPC marker NG2, and oligodendrocyte marker MBP (Supplementary Fig. S1b, c). Similarly, after culturing the GFP-positive astrocytes in NIM for 8 days, 30% GFP-expressing cells were positive for DCX, Tuj1 and MAP2, and 16% cells were positive for NeuN (Fig. 1e, g). These data suggest that neonatal astrocytes isolated from the cortex region can be converted into neuronal cells in neurogenic differentiation culture medium without further chemical addition.

### Neuronal cells were induced from neonatal astrocytes, but not NPCs

**Fig. 2:**
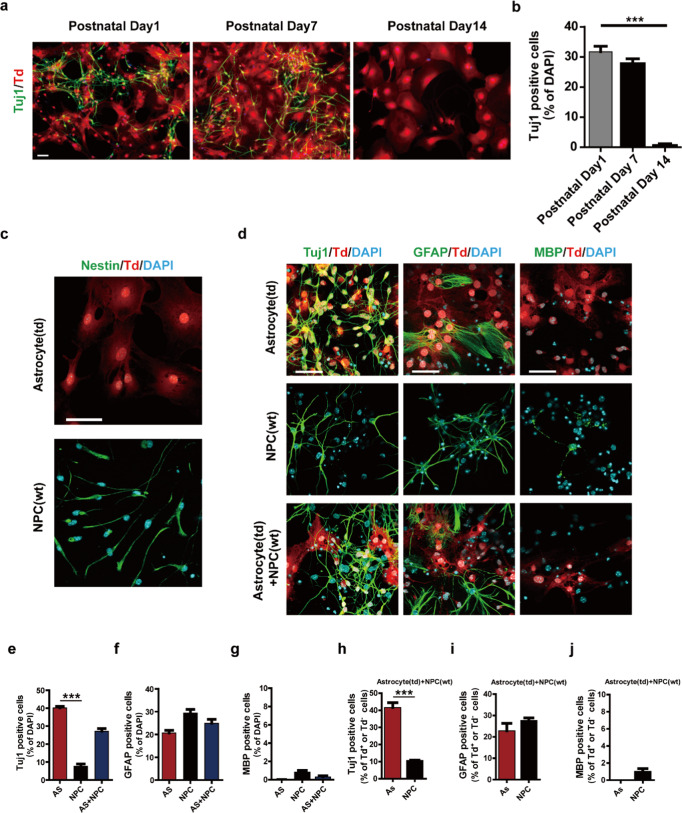
Neuronal cells were originated from neonatal astrocytes, but not from NPCs. **a** Immunostaining of cells derived from day 1, day 7 or day 14 postnatal astrocytes cultured in NIM on day 8 with Tuj1 antibodies. **b** Statistic quantification of **2a** (mean ± SEM, *n* = 3 independent experiments). ***P < 0.001 vs postnatal day 1. **c** Characterization of cultured GFAP-Cre:R26tdTomato mouse cortical astrocytes and cortical NPCs from wild type mice. Nestin was stained as green with a Nestin antibody and nuclei were visualized by DAPI. **d** Immunostaining of astrocytes, NPCs and mixed co-cultures in induction medium on day 8 with Tuj1, GFAP and MBP antibodies. **e**–**g** Quantification of neuron (**e**), astrocyte (**f**) and oligodendrocyte (**g**) percentage within total cells as shown in **d** (mean ± SEM, *n* = 3 independent experiments). ***P < 0.001 vs AS. **h**–**j** Statistic analysis of lowest panel in **d**. Percentage quantification of tdTomato positive neuron (**h**), astrocyte (**i**) and oligodendrocyte (**j**) derived from tdTomato positive astrocytes or tdTomato negative neuron (**h**), astrocyte (**i**) and oligodendrocyte (**j**) from tdTomato negative NPCs in the co-cultures (mean ± SEM, *n* = 3 independent experiments). ***P < 0.001 vs AS. Scale bars, 50 μm

Next, we examined the conversion ability of astrocytes isolated from the cortices of mice at different postnatal stages. Neural conversion with NIM was observed when using day 7 postnatal astrocytes but not day 14 (Fig. 2a, b). The result demonstrated that neuronal conversion ability exists in early postnatal astrocytes, which led to two questions: (1) Was it possible that the neural conversion we observed was actually from contaminated NPCs, although we did not detect NPC marker in the astrocyte culture (Fig. 1b, Supplementary Fig. S1a); (2) Did the neonatal cortical astrocytes resemble NPCs? To address these questions, comparison studies were carried out using NPCs and astrocytes isolated from neonatal mouse cortex. Immunofluorescence staining showed that Nestin were expressed in NPCs, but not in astrocytes (Fig. 2c), and the cells displayed distinct morphologies. After culturing astrocytes and NPCs in the same NIM for 8 days, different results were observed (Fig. 2d, upper and middle panel). Up to 40% of the cells were Tuj1-positive in astrocyte cultures, while only 7.6% of the cells were Tuj1-positive when using NPCs as the starting cells (Fig. 2e). In addition, the neuronal cells derived from astrocytes and NPCs present different morphologies. Furthermore, only 20% of the tdTomato-positive cells maintained GFAP-positive and tile shape at day 8 (Fig. 2d, f) and no MBP-positive cells were detected in astrocyte cultures (Fig. 2g), whereas in the NPC culture, 30% of the cells became GFAP-positive and stellate shape, and 1% became MBP-positive (Fig. 2d, middle panel and 2f-g). Considering the pure NPC culture generated much lower number of Tuj1-positive neurons than the astrocyte culture, it was very likely that the neurons we observed in astrocyte culture were indeed converted from astrocytes.

To rule out the remote possibility that the contaminated NPCs in astrocyte culture may grow faster due to the presence of astrocytes [24], we designed an experiment to co-culture tdTomato-positive astrocytes and tdTomato-negative NPCs with a 1:1 ratio in the neural induction condition for 8 days. Analysis of the co-culture showed that about 27.1% of the total cells were positive for Tuj1, 24.8% for GFAP, and 0.3% for MBP (Fig. 2d, lowest panel and 2e–g), reflecting a compromised performance of the two types of mixed cells. Interestingly, when tracing the cell origination, the ratio of Tuj1-, GFAP- and MBP-positive cells among tdTomato-positive cells were 41.7%, 22.8%, and 0%, respectively, very similar to the pure astrocyte culture; while the ratios of Tuj1-, GFAP- and MBP-positive cells among tdTomato-negative cells were 10.6%, 27.7% and 1.3%, respectively, also similar to the pure NPC culture (Fig. 2h–j). These data suggest that the neuronal cells indeed originate from cortical astrocytes, but not NPCs, and cortical astrocytes and NPCs have distinct neuronal conversion potential and do not resemble each other.

### Insulin in the NIM is essential for the astrocyte-to-neuron conversion

**Fig. 3:**
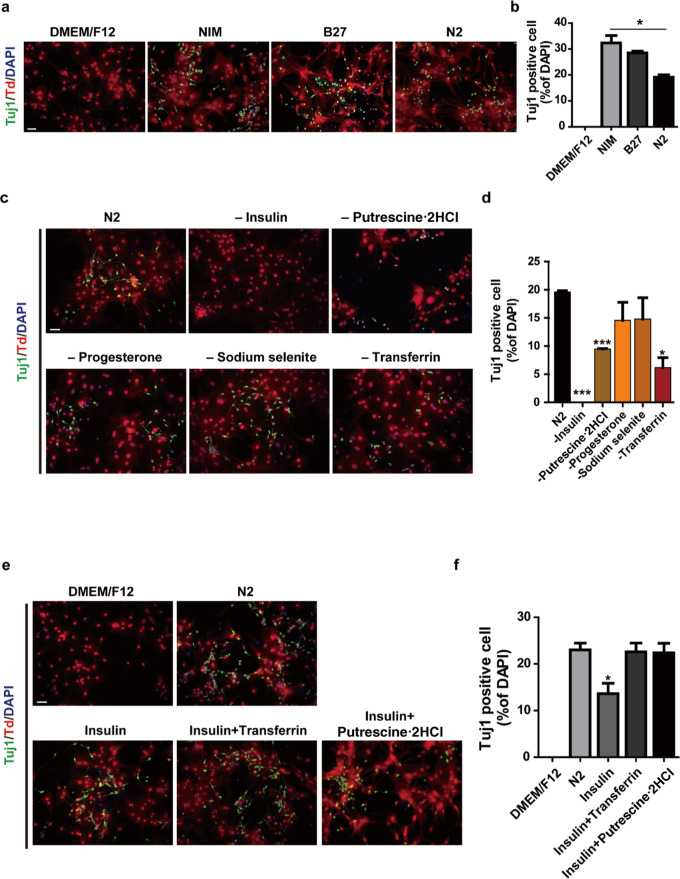
Insulin in the medium is essential for the astrocyte-neuron conversion. **a** Cells were cultured in DMEM/F12 basic medium, NIM, B27 or N2 supplemented medium for 8 day and subjected to immunostaining with the Tuj1 antibody. **b** Quantification of Tuj1 positive cell percentage within total cells as shown in **a** (mean ± SEM, *n* = 3 independent experiments). *P < 0.05 vs NIM. **c** Immunostaining of tdTomato positive cells cultured in N2 medium or N2 medium removing one of N2 supplement component each time for 8 days with Tuj1 antibody. **d** Quantification of Tuj1 positive cells percentage within total cells as shown in **c** (mean ± SEM, *n* = 3 independent experiments). *P < 0.05 or ***P < 0.001 vs N2. **e** Immunostaining of tdTomato positive cells cultured in DMEM/F12 basic medium, N2 medium and DMEM/F12 basic medium adding insulin, insulin and transferrin, insulin and putrescine·2HCl separately for 8 days with Tuj1 antibody. **f** Quantification of Tuj1 positive cells percentage within total cells as shown in **e** (mean ± SEM, *n* = 3 independent experiments). Scale bars, 50 μm. *P < 0.05 vs N2

Given that the astrocyte-to-neuron conversion was achieved without extra chemical addition, we wondered which component in the NIM contributed to the conversion. NIM is comprised of DMEM/F12, 2% B27, 1% N2, neurotrophic factors GDNF (20 ng/mL) and BDNF (20 ng/mL). We first compared the media supplemented with only B27 or N2. As expected, no Tuj1-positive cells could be seen in DMEM/F12 basal medium, and approximate 30% Tuj1-positive cells were detected in NIM. Interestingly, the ratio of Tuj1-positive cells was ~28% in medium containing B27, and ~19% in medium supplemented with N2 alone (Fig. 3a, b).

Since N2 supplement contains mainly five components: insulin, transferrin, sodium selenite, progesterone and putrescine·2HCl, which are also found in B27 supplement. We wanted to find out which molecule is the key factor to induce astrocyte to neuron conversion by removing one of those components each time. As shown in Fig. 3c, d, removal of sodium selenite or progesterone did not hinder the conversion significantly. When astrocytes were cultured in medium without transferrin or putrescine·2HCl, the conversion rate was significantly decreased. Interestingly, when insulin was removed, very few Tuj1-positive cells could be observed. These results indicate that insulin is indispensable for the conversion, and transferrin and putrescine also contribute to the glia-to-neuron conversion process. We further reconstituted the supplement with insulin and transferrin or putrescine·2HCl. In consistent with previous observations, insulin alone rescued nearly 70% of the conversion rate (13.6% Tuj1-positive cells) compared to that of N2 (20% Tuj1-positive cells), whereas insulin combined with either transferrin or putrescine·2HCl was sufficient to resemble the effect of N2 (Fig. 3e, f). All these results suggested that insulin is essential for the astrocyte-to-neuron conversion.

### Neuronal cells derived from neonatal cortical astrocytes showed functional maturation

**Fig. 4:**
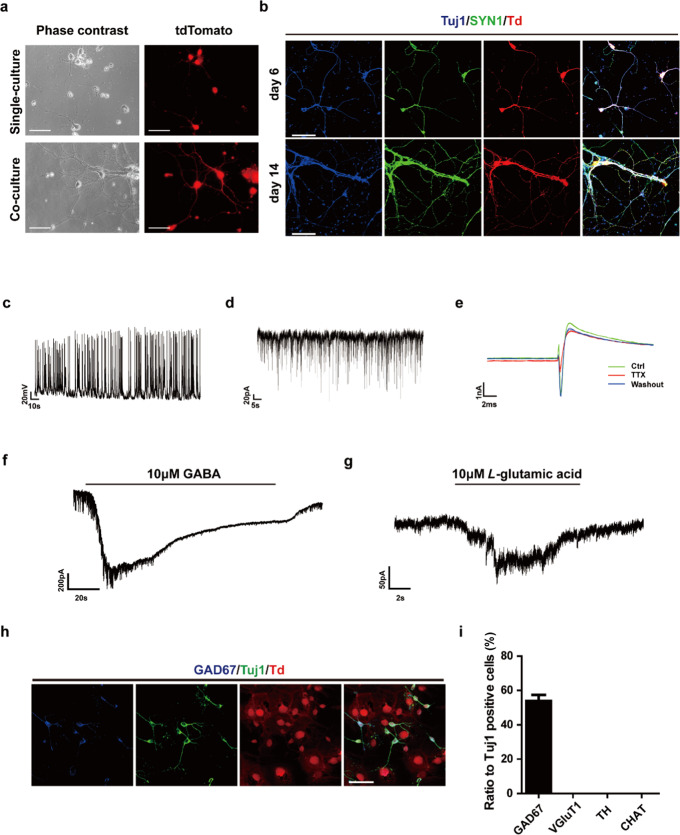
Functional maturation of induced neuronal cells. **a** Induced cells displayed more mature neuronal morphology after co-cultured with primary neurons. **b** Induced cells expressed mature neuronal markers SYN1. **c** Representative traces of repetitive action potentials recorded on induced tdTomato positive neuronal cells on day 14. **d** Representative traces of spontaneous postsynaptic currents on induced tdTomato positive neuronal cells. **e** Representative inward sodium currents on induced neuronal cells. The inward currents could be blocked by Na^+^ channel blocker tetrodotoxin (TTX) and regained when TTX was washed out. **f** Focal application of GABA induced inward membrane currents. **g** Focal application of *L*-glutamic acid induced inward membrane currents. **h** The induced neuronal cells were mainly GAD67 positive GABAnergic neurons. **i** Quantification of GABAnergic neurons percentage as shown in **h** (mean ± SEM, *n* = 3 independent experiments). Scale bars, 50 μm

Next, we evaluated whether the astrocyte derived neuronal cells had functional maturity. To promote the maturation, induced neuronal cells were re-plated after a 4-day induction onto a pre-existing monolayer culture of primary neurons [25]. The tdTomato positive neuron-like cells exhibited more mature neuronal morphology with longer axons and dendrites after a 10-day co-culture period (Fig. 4a). Immunostaining revealed that on day 6 of the co-culture, the tdTomato positive cells were already double positive for Tuj1 and the mature neuronal marker Syn1; on day 14, the cells formed even more complex neuronal networks (Fig. 4b).

We examined the electrophysiological properties of the tdTomato-positive neuronal cells by whole-cell patch-clamp. These cells generated spontaneous repetitive trains of action potentials (APs) (Fig. 4c) and showed typical spontaneous postsynaptic currents (sPSCs) (Fig. 4d). Significant sodium currents were detected, which can be blocked by the sodium channel blocker tetrodotoxin (TTX) and recovered when TTX was washed out (Fig. 4e). Inward currents were induced when exogenous GABA or *L*-glutamic acid were puffed onto cells, indicating that functional glutamate and GABA receptors were present in these neuronal cells (Fig. 4f, g). These data demonstrate that the neuronal cells derived from mouse neonatal cortical astrocytes are functional.

We also tried to identify subtypes of neurons induced from neonatal cortex astrocytes. Immunostaining results showed that approximately 54% of the converted neurons were positive for GABAergic neuron marker GAD67, while no positive signal was detected for glutamatergic neuron marker VGluT1, dopaminergic neuron marker TH or cholinergic neuron marker CHAT (Fig. 4h, i), suggesting that the converted neuronal cells are mainly GABAergic.

### Induced neuronal cells can survive and mature in the mouse brain

**Fig. 5:**
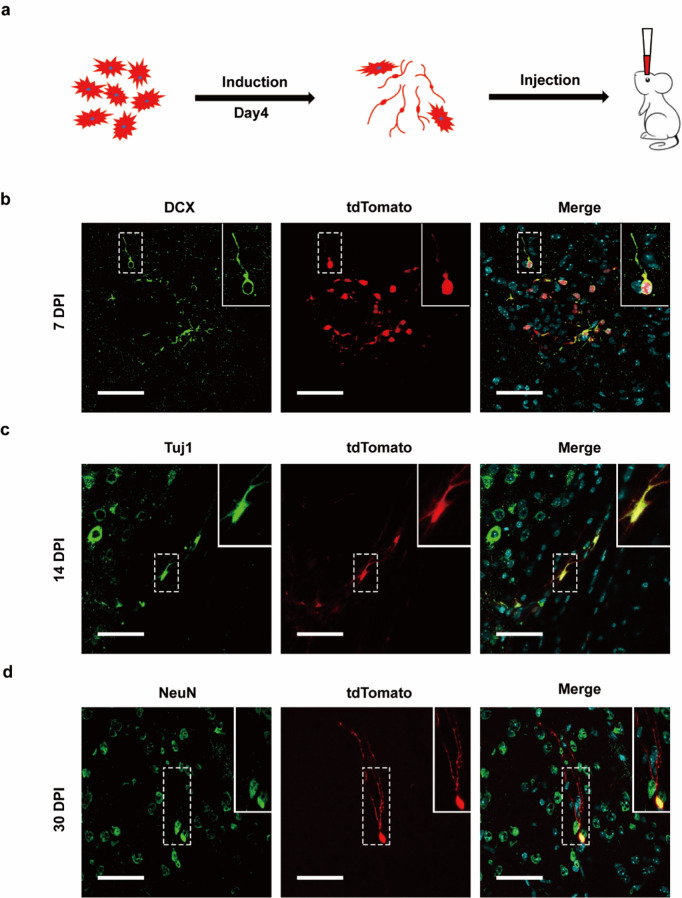
Survival of induced neuronal cells in mouse brains. **a** Schematic drawing showing the transplantation of converted neurons into the mouse brains. **b** tdTomato^+^ cells were identified around cortex on 7 day post cell injection (DPI). Many tdTtomato^+^ cells were positive for DCX. **c** On 14 DPI, some tdTomato^+^ cells were immunopositive for Tuj1. **d** On 30 DPI, some tdTomato^+^ cells were immunopositive for NeuN. Scale bars, 50 μm

We next investigated the survival and maturation of the astrocytes derived neuronal cells by tracing the cells via tdTomato signal in vivo. After culturing tdTomato labeled astrocytes in NIM for 4 days, the derived cells were harvested and injected into the lateral ventricle of neonatal wildtype mice (Fig. 5a). At 7th day post cell injection (DPI), tdTomato positive cells showed neuronal morphology; and were positive for DCX and Tuj1 staining on day 14 (Fig. 5b, c). In addition, mature neuronal marker NeuN was detectable in these cells on day 30 and a mature neuron morphology with a complex network of neurites was observed (Fig. 5d). These results suggest that the astrocyte derived neuronal cells can survive and mature in the mouse brain.

### Astrocytes from different brain regions possess various potential in neuronal conversion

**Fig. 6:**
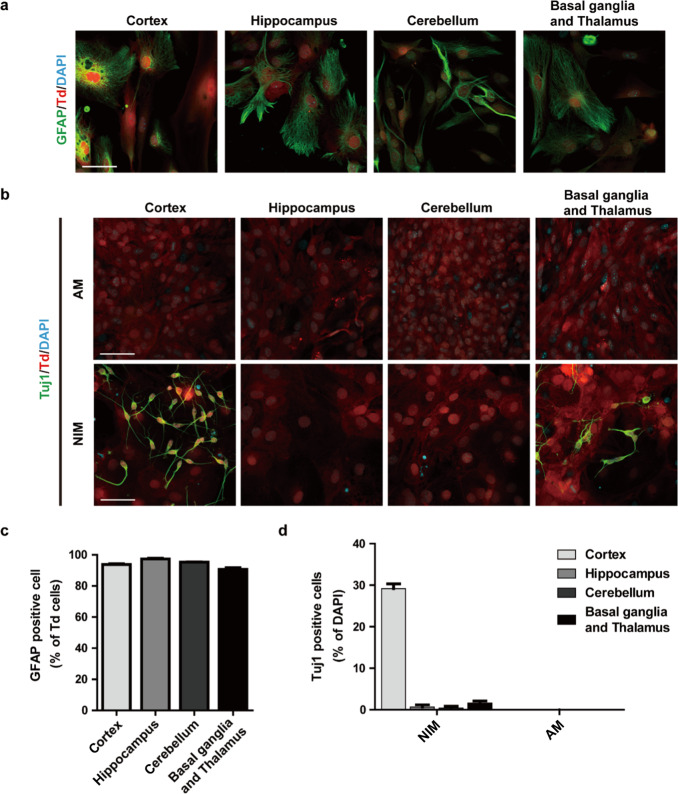
Comparison of the neuronal conversion potential of astrocytes from different brain regions. **a** Immunostaining of astrocytes from different CNS regions with GFAP antibodies. **b** Immunostaining of astrocytes from different CNS regions cultured in AM or NIM  for 8 days with Tuj1 antibodies. **c** Quantification of **a** (mean ± SEM, *n* = 3 independent experiments). **d** Quantification of **b** (mean ± SEM, *n* = 3 independent experiments). Scale bars, 50 μm

The above results suggest that astrocytes from neonatal cortical region of mouse brain can be easily converted to neurons by culturing in a commonly used medium. To further investigate whether astrocytes derived from different regions of the brain have similar potential, tdTomato-positive astrocytes were isolated from cortex, hippocampus, cerebellum and the rest of forebrain (Basal ganglia and Thalamus). Nearly all these astrocytes were double positive for tdTomato and GFAP (Fig. 6a, c). After 8 day culture in NIM, abundant Tuj1 positive cells (29.0%±2.2%) were detected in cortex derived astrocyte culture; while the ratio of Tuj1 positive cells was only 0.7% in the hippocampus astrocyte, 0.4% in the cerebellum astrocyte and 1.6% in the basal ganglia and thalamus astrocyte cultures (Fig. 6b, d). When astrocytes were maintained in AM, neuronal markers can be hardly detected at day 8. These data indicated that only cortex astrocytes possess a pronounced ability of neuronal conversion.

## Discussions

Previous studies have shown that GFAP-expressing astrocytes in the adult subventricular zone or subgranular zone can function as neural stem cells [12, 13]. In the current study, we found that neonatal mouse cortical astrocytes had the intrinsic ability to turn into neuronal cells in vitro which differed from that seen in NPCs.

Since GFAP expression is debatable for its specificity in astrocytes [26], we used another astrocyte specific marker ALDH1L1 [27] to trace astrocytes and observed the same phenomenon, supporting our conclusion that the neuronal cells are indeed originated from the astrocytes in our studies. Besides, this conversion can be easily detected within the early stage of neonatal astrocytes, and the efficiency dropped dramatically when using astrocytes isolated from elder mice (Fig. 2a). Coincidentally, previous study has shown that the ability to form neurospheres is restricted to astrocytes obtained during the first two postnatal weeks [28]. These observations suggest that there might exist a small population of neonatal cortical astrocytes possessing some properties of NPCs which could not be preserved very well along with the individual growing up. It is known that radial glia cells are the main source of neurons during embryogenesis and shift exclusively towards astrocyte generation after neurogenesis [29–33]. Therefore, it is reasonable to speculate that the population within postnatal astrocytes is at an intermediate stage between radial glia and mature astrocyte, and still latently retain some of radial glia properties for neurogenesis.

Previous studies showed widespread localization of insulin receptor in brain areas important for mood and cognition, including the hypothalamic and hippocampal regions [34, 35]. Since 1979 when Bottenstein and Sato defined a serum free medium for neural cell culture, insulin has been supplied as a standard component within N2 or B27 for the serum replacement [36]. Insulin-like growth factor I (IGF‑I) and insulin-like growth factor II (IGF‑II) are synthesized in the brain and their levels decline with ageing, and insulin and IGF receptor signaling have been reported to be important in neural‑stem-cell homeostasis. IGF‑I enhances proliferation and/or differentiation of diverse stem-cell populations in rodents and humans, including embryonic stem cells, NSCs and mesenchymal stem cells; and IGF-II have been described for its role in NSC maintenance and neural progenitor expansion [37]. Inhibition of brain insulin signaling results in neuroplasticity deficits, including aberrant hippocampal glutamatergic transmission and impaired long-term potentiation [38]. So far, only a few studies have investigated the role of insulin in astrocyte functions. A recent study demonstrated that astrocytes are a direct insulin target in the brain and that knockout of IR on astrocytes causes increased anxiety- and depressive-like behaviors in mice. At a molecular level, loss of insulin signaling in astrocytes impaired tyrosine phosphorylation of Munc18c, which led to decreased exocytosis of ATP from astrocytes, resulting in decreased purinergic signaling on dopaminergic neurons [39]. Reduced IGF-I signaling in astrocytes impairs their support for neurons under conditions of stress which is associated with defects in the mitochondrial respiratory chain in astrocytes [40]. Very interestingly, we found in the current report that insulin is essential for neonatal cortical astroglia-to-neuron conversion. And insulin combined with either transferrin or putrescine could resemble the effect of N2 supplement. We also noticed recent publication in which isolated mouse astrocytes were converted to functional neurons by depleting the RNA-binding protein PTB, and PTB promotes insulin expression via binding to the untranslated region of insulin mRNA [41, 42] and insulin secretory granule biogenesis [43]. In order to test whether PTB is involved in our study, we compared the expression of *PTB* mRNA among different astrocytes. The results show that astrocytes express a higher level of *PTB* than neurons, however, there is no clear correlation between the *PTB* expression in astrocytes from different brain regions and their potential in neuronal conversion (data not shown), which suggests that *PTB* expression might not be involved in the astrocyte-to-neuron conversion phenomenon we observed, the underlying mechanisms still require further elucidation.

As mentioned, astrocytes are a diverse population with morphological and functional heterogeneity [44–47]. Our results revealed a distinct subtype of astrocytes with intrinsic neuronal conversion ability. We also observed the distinguishing neuronal conversion potential between astrocytes derived from cortex and hippocampus, in consistent with the current view that different brain regions harbor astrocytes with different functional properties. Yet, the astrocyte morphology does not clearly correlate with function [45], so it is feasible to seek the molecular difference responsible for the unique function in different astrocyte population [48, 49]. We investigated the differentially expressed genes in cortical and hippocampus astrocytes by transcriptome sequencing. Subsequent bioinformatic analysis revealed that the main differences are located in genes involved in chromosome segregation, mitotic nuclear division and DNA replication. We also intend to explore the particular molecular markers of astrocyte with neuronal differentiation potential using 10**×** genomic single cell RNA-seq. Gene ontology analysis also revealed that one cluster of astrocytes was significantly enriched in RNA splicing, chromosome segregation and regulation of cell cycle process. Interestingly, a previous report found that cortex astrocytes proliferated faster showed higher neuronal reprogramming efficiency than astrocytes from cerebellum and spinal cord [18]. Coincidentally, when tracing the neuronal induction process from astrocytes, we observed prominent cell division in cells with apparent morphological changes towards neuronal cells (data not shown). All these evidences imply that the neuronal conversion ability of cortical astrocytes is probably related to their proliferation ability. In line with these indications, we found genes, such as KIF2C, TOP2A and HMMR which are related to cell proliferation [50–52], were expressed in one cluster of cortical astrocytes with relative high level. Whether those genes may contribute to the neuronal conversion events needs to be further elucidated.

Altogether, our findings suggest that neonatal astrocytes from certain brain regions possess intrinsic potential to differentiate/transdifferentiate into neurons which may have clinical relevance in the future.

## Supplementary information

Supplementary Information

Supplementary Video 1
