# Supplementary material for: Neonatal cortical astrocytes possess intrinsic potential in neuronal conversion in defined media
Source: Acta Pharmacol Sin. 2021 Feb 5;42(11):1757–68. doi: 10.1038/s41401-020-00586-0 (PMC8563807; doi:10.1038/s41401-020-00586-0)
Supplement: Supplementary file 1 — Supplementary Information [file 41401_2020_586_MOESM1_ESM.docx]

**Legends**

**Fig. S1** **Characterization of cultured neonatal mouse cortical astrocytes**

**a** Characterization of cultured GFAP-Cre:R26tdTomato mouse cortical astrocytes.

**b** Characterization of cultured ALDH1L1:GFP mouse cortical astrocytes.

**c** Quantification of cell percentage positive for astrocyte markers (GFAP, S100β), neuronal markers (DCX and Tuj1), oligodendrocyte marker (MBP), and NG2 glial marker (NG2) in cultured ALDH1L1:GFP mouse cortical astrocytes as shown in **S1b** (mean ± SEM, n = 5 independent experiments). Scale bars, 50 μm.

**d-k** qPCR analysis of gene expression in isolated cells: representative astrocyte specific gene *GFAP* **(d)** *Aldoc* **(g)**, *GS* **(h)**and *CD44***(i)**, NPC specific gene *Nestin* **(e)** and *Sox2* **(j)**, neuron specific gene *Tuj1* **(f)** and *NeuN* **(k)**.

**Fig. S2** **Live image of morphological changes of astrocytes in the neuronal converting process of Video 1.** Live images were captured for 8 days and one representative frame from each day were selected. White arrows indicated the cells which were converted to neuronal cells. Scale bars, 50 μm.

**Fig. S3** **Induction of neuronal cells from tdTomato positive astrocytes.**

**a-d** Immunostaining of tdTomato positive cells with DCX **(a)**, Tuj1 **(b)**, MAP2 **(c)** and NeuN **(d)** antibodies respectively. Scale bars, 50 μm.

**Supplementary Video 1 Live image of morphological changes of astrocytes in the neuronal converting process related to FigS2.** Live images were captured for 8 days using Olympus IX81-ZDC2 microscope.

**Supplementary Table S1 Primer sets for qPCR reactions.**Figure S1
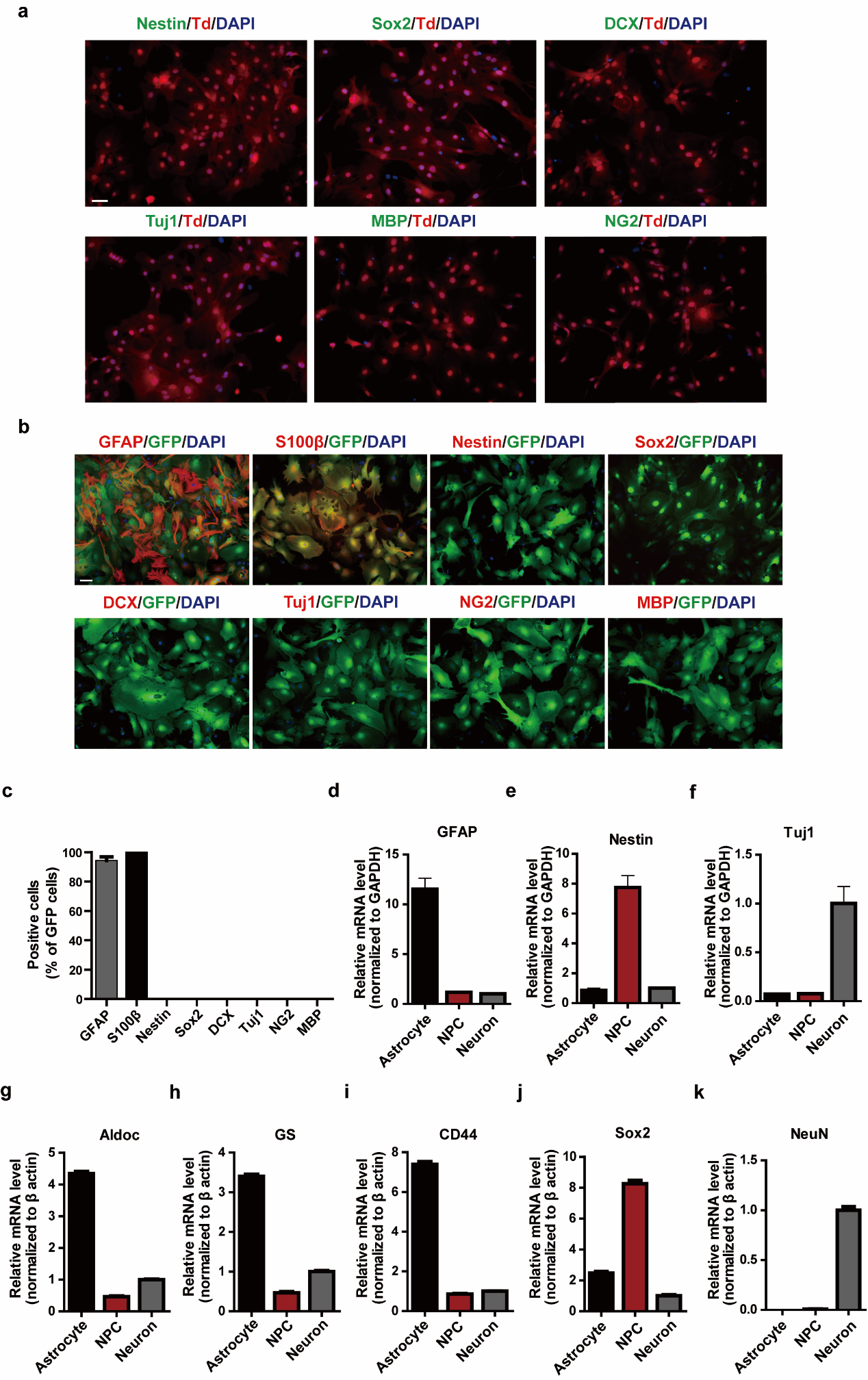


Figure S2


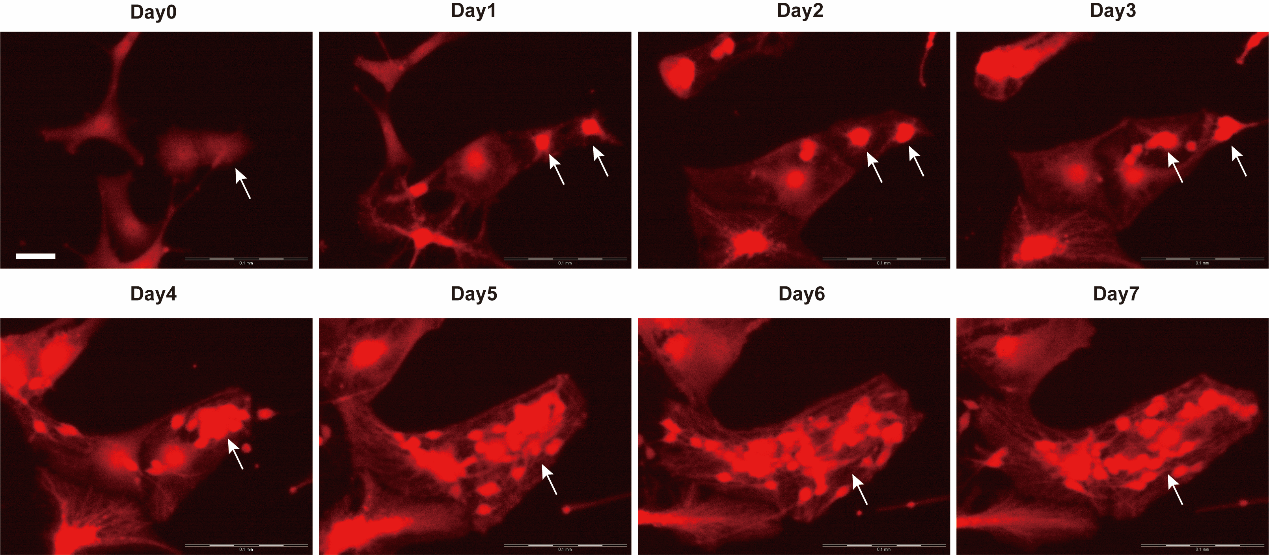


Figure S3


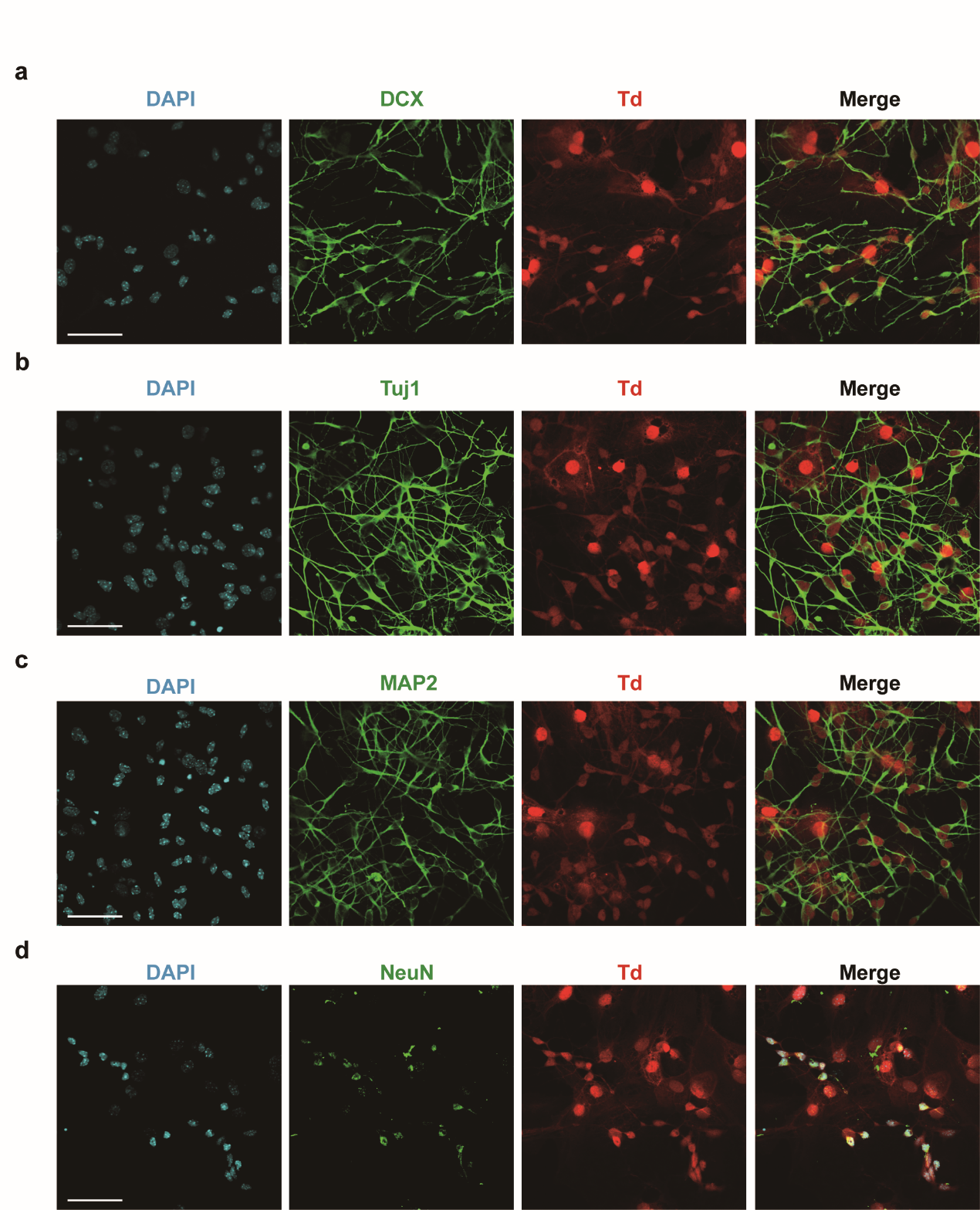


Supplementary Table S1

| **Gene** | **Sequence** | **(5’-3’)** |
| --- | --- | --- |
| ***GFAP*** | Forward | GAAACCAACCTGAGGCTGGA |
|  | Reverse | CCACATCCATCTCCACGTGG |
| ***Aldoc*** | Forward | CGGAGACCATGACCTCAAAC |
|  | Reverse | GGTCACTCAGGGCCTTGTAT |
| ***CD44*** | Forward | TCATCCCAACGCTATCTGTGC |
|  | Reverse | TCTATACTCGCCCTTCTTGCTGTA |
| ***GS*** | Forward | TCCCACTTGAACAAAGGCATC |
|  | Reverse | CACTCAGGTAACTCTTCCACACACT |
| ***Nestin*** | Forward | TGAGGCTCCCTATCCTAAAAATG |
|  | Reverse | CTAAAATAGAGTGGTGAGGGTTGAG |
| ***Tuj1*** | Forward | CGTCAAGGTAGCCGTGTGTG |
|  | Reverse | TTCCGATTCCTCGTCATCATC |
| ***NeuN*** | Forward | AGACAGACAACCAGCAACTCCA |
|  | Reverse | CCCCGCTCGTTAAAAATGAT |
| ***Ptbp1*** | Forward | GTCCCAGACATAGCAGTCGGTA |
|  | Reverse | CTTCAGCATGAGAAGGTTGGTAAC |
| ***Actin*** | Forward | GGCTGTATTCCCCTCCATCG |
|  | Reverse | CCAGTTGGTAACAATGCCATGT |
| ***GAPDH*** | Forward | GTGTTCCTACCCCCAATGTGT |
|  | Reverse | ATTGTCATACCAGGAAATGAGCTT |
